# High basal Wnt signaling is further induced by PI3K/mTor inhibition but sensitive to cSRC inhibition in mammary carcinoma cell lines with HER2/3 overexpression

**DOI:** 10.1186/s12885-015-1544-y

**Published:** 2015-07-25

**Authors:** Elpetra P. M. Timmermans-Sprang, Ana Gracanin, Jan A. Mol

**Affiliations:** Department of Clinical Sciences of Companion Animals, Faculty of Veterinary Medicine, Utrecht University, Yalelaan 104, 3584 CM Utrecht, The Netherlands

**Keywords:** Wnt signaling, PI3K/AKT/mTOR, cSRC, PTEN, HER2/3, Mammary cancer, Canine

## Abstract

**Background:**

Elevated basal, ligand-independent, Wnt signaling in some canine breast cancer cells is not caused by classical mutations in APC, β-Catenin or GSK3β but, at least partially, by enhanced LEF1 expression. We examined the expression and function of EGFR/HER-regulated pathways on the ligand-independent Wnt signaling.

**Methods:**

Twelve canine mammary tumor cell lines with previously reported differential basal Wnt activity were used. The expression levels of genes related to EGF-signaling were analyzed by cluster analysis. Cell lines with a combined overexpression of EGF-related genes and enhanced basal Wnt activity were treated with PI3K/mTor or cSRC inhibitors or transfected with a construct expressing wild-type PTEN. Subsequently, effects were measured on Wnt activity, cell proliferation, gene expression and protein level.

**Results:**

High basal Wnt/LEF1 activity was associated with overexpression of *HER2/3*, *ID1*, *ID2*, *RAC1* and *HSP90* together with low to absent *cMET* and *PTEN* mRNA expression, suggesting a connection between Wnt- and HER-signaling pathways. Inhibition of the HER-regulated PI3K/mTor pathway using the dual PI3K/mTor inhibitor BEZ235 or the mTor inhibitor Everolimus® resulted in reduced cell proliferation. In the cell line with high basal Wnt activity, however, an unexpected further increased Wnt activity was found that could be greatly reduced after inhibition of the HER-regulated cSRC activity. Inhibition of the PI3K/mTor pathway was associated with enhanced expression of *β-Catenin*, *Axin2*, *MUC1*, *cMET*, *EGFR* and *HER2* and a somewhat increased β-Catenin protein content, whereas cSRC inhibition was associated with slightly enhanced *HER3* and *SLUG* mRNA expression. A high protein expression of HER3 was found only in a cell line with high basal Wnt activity.

**Conclusions:**

High basal Wnt activity in some mammary cancer cell lines is associated with overexpression of HER-receptor related genes and HER3 protein, and the absence of *PTEN*. Inhibition of the PI3K/mTor pathway further stimulated, however, canonical Wnt signaling, whereas the inhibitory effect with the cSRC inhibitor Src-I1 on the Wnt activity further suggested a connection between Wnt and HER2/3-signaling.

## Background

Progesterone-induced Wnt signaling plays an important role in the development of the mammary gland during puberty, but may also induce the development of mammary tumors from stem/progenitor cells [1]. There is a high incidence of active Wnt signaling in basal-like breast cancers [2]. Since this activity cannot always be explained by overexpression of Wnt ligand or mutations in players involved in the destruction complex of β- Catenin alone [3], this high Wnt signaling must have additional causes. One potential candidate, cytoplasmic β-Catenin, functions as a key signaling intermediate in the canonical Wnt/β-Catenin pathway. Wnt proteins, by binding to membrane receptors on the cell membrane, initiate the signal transduction. The total Wnt group consists of multiple genes that are either canonical (Wnt 1, 3A, 8A, 8B) or non-canonical (Wnt 5A, 4, 11). Wnt can also stimulate alternative effectors in the non-canonical pathway that can antagonize the canonical pathway [3].

Without a Wnt signal, the β-Catenin destruction complex binds and phosphorylates non-cadherin-associated β-Catenin. This targets it for destruction by the proteasome, thereby allowing TCF family of transcriptional factors to bind to transcriptional repressors in the nucleus. The destruction complex is disrupted when active Wnt signaling is stimulated by binding of Wnt proteins to its Frizzled receptor on the cell membrane. As a result β-Catenin accumulates in the cytoplasm, translocates to the nucleus and associates with TCF family of proteins, enhancing transcriptional activation of a program of genes [3–5].

In the non-canonical Wnt pathway, Wnt can act through ROR and RYK tyrosine kinases, which activate Jun N-terminal kinase (JNK). This increases intercellular Ca^2+^ leading to activation of nuclear factor activated T cells (NFAT) and consequently inhibition of the canonical signaling. The alternative Wnt receptor RYK additionally signals through Dishevelled to cSRC in axon guidance. Traditionally Wnt ligands do not signal through β-Catenin in the non-canonical pathway, but it seems that Wnt 5A had some inhibitory characteristics on the canonical signaling. Likely there is some crosstalk between the canonical and the non-canonical Wnt pathways [4].

The major drivers of breast cancer risk are 17ß-estradiol (E2) and progesterone (P4). Synthetic progestins are frequently given as oral anticonception or in hormone replacement therapy (HRT) [6, 7]. Apart from stimulating the expression of Wnt4 [3] P4 decreases the presence of the ß-catenin binding adhesion protein E-cadherin, which may result in higher Wnt/β-Catenin signaling [8]. P4 also affects the non-canonical signaling through the transcriptional activity of activator protein 1(AP1) in the signaling cascade of JNK and MAPK [9]. In the dog, P4 also plays an important role in mammary cancer [10]. This makes dogs a useful model, since they share human environmental risk factors and their mammary tumor biology shows a significant overlap with humans: sequencing canine simple mammary carcinomas revealed comparable genomic aberrations to those in human breast cancer. Further, both humans and dogs apparently share pathways that are altered in carcinogenesis, such as cell adhesion, Wnt signaling and PI3K signaling [11], making them an attractive model for hormone-dependent breast cancer research [5, 12].

Recently we have shown that three canine mammary tumor cell lines with high basal Wnt/β-Catenin activity do not respond to treatment with the Wnt ligand synthesis inhibitor IWP-2 and therefore use a ligand-independent mechanism to activate the pathway. In 4 other cell lines, moderate basal Wnt reporter activity could be inhibited using IWP-2 showing a clear ligand depend Wnt activity, the remaining cell lines had no basal Wnt activity. We furthermore showed that overexpression of LEF1 is one of the contributing factors of the high Wnt/β-Catenin activity in these cells [5]. In this study we demonstrate that these cells also have lost *PTEN* expression. PTEN is a tumor suppressor gene and a phosphatase that antagonizes the kinase activity of PI3K. PTEN can also target focal adhesion kinase (FAK), the EGF receptor and itself as a binding partner to increase p53 activity [13]. A properly functioning PTEN thus inhibits PI3K/AKT/mTOR and MAPK signaling.

The epidermal growth factor (EGF) is transactivated by the Wnt pathway, which in addition stimulates the ß-catenin/TCF pathway, making Wnt a potent oncogene in the mammary gland [14]. Binding of EGF or related growth factors to the EGF receptors induces homo- and heterodimers leading to phosphorylation on specific tyrosine residues; these residues serve as docking sites for a variety of signaling molecules, leading to activation of intracellular pathways such as the mitogen-activated protein kinase (MAPK), the phosphatidylinositol-3-kinase (PI3K), Stats, RAS and cSRC pathways. Although not binding any ligand, HER2 plays a central role together with the HER3 protein that lacks proper tyrosine kinase activity, with this complex being the strongest activator for downstream signaling pathways [14, 15]. HER3 can also signal ligand independent and its activation is associated with resistance to HER2 targeting tyrosine kinase inhibitors in breast cancer [16]. The HER3 protein, which has no kinase activity, may signal in the nucleus through several C-terminal transactivation domains [17]. Also in the dog, HER2 is overexpressed in some 35 % of malignant mammary tumors whereas HER3 is found in the nucleus of some 42 % of mammary carcinomas [18].

We therefore investigated in a panel of canine mammary tumor cell lines for a relationship between the canonical Wnt signaling and HER signaling pathways. As shown recently these cell lines varied in basal Wnt/ß-Catenin signaling from high ligand-independent to moderate ligand-dependent or absent basal [5].

## Methods

### Canine mammary cell lines and culture

Canine mammary tumor cell lines used in this study were CMT1, CMT-U229, CMT-U335, CMT-U27, CMT9, P114, CHMp, CHMm, CNMp, CNMm, CIPp and CIPm [19–21]. The cell lines were generous gifts of the Prof Dr Hellmen (SLU, Uppsala, Sweden), Prof Dr Sasaki (Laboratory of Veterinary Surgery, University of Tokyo, Japan), and Dr Rutteman (Utrecht University, The Netherlands). All cell lines were cultured in DMEM/F12 (Invitrogen, Bleiswijk, The Netherlands) supplemented with 10 % fetal bovine serum (FBS) (FBS Gold, PAA, Cӧlbe, Germany). Cells were tested to be free from mycoplasma with a Mycosensor QPCR assay according to manufacturer’s protocol (Agilent technologies, Middelburg, The Netherlands).

### TCF-reporter assay

Cells were seeded in a 24 well plate (Primaria, BD Biosciences, Breda, The Netherlands) at a density of 100,000 CMT1, CMT-U27 and CMT9 cells and 80,000 CIPm cells, to reach an 80 % density 24 h before transfection. Transfection was performed in FBS-free medium using 3 μl Lipofectamine 2000 (Invitrogen), 800 ng pTOPFLASH (TOP) or pFOPFLASH (FOP) (gift from Prof Dr Hans Clevers, Hubrecht Institute, The Netherlands) and 0.5 ng human ß-actin-promoter renilla construct [22] as an internal control. Transfection was stopped after 5 h by adding the same volume DMEM/F12 supplemented with 20 % FBS. Cells were treated with 100 nM Everolimus (Selleckchem, Munich, Germany), 50 nM BEZ235, (Selleckchem), 20 μM Src-I1 (Enzo, Lausen, Zwitserland), or 1 μM FAK Inhibitor 14, (Santa Cruz, Heidelberg, Germany) for 40 h. All the compounds were dissolved in DMSO and diluted in medium to a final concentration of 0.2 % DMSO. The firefly and renilla luciferase activities were measured using a Dual-Luciferase Assay System (Promega, Leiden, The Netherlands) in a Centro LB 960 luminometer (Berthold Technologies, Vilvoorde, Belgium).

### Real time quantitative RT-PCR

From each cell line, total RNA was isolated and treated with DNase using RNeasy mini kit (Qiagen, Venlo, The Netherlands) according to manufacturer’s protocol. Using iScript kit (BioRad, Veenendaal, The Netherlands), cDNA synthesis was performed. Specific primer sets were used to amplify gene products in a qPCR reaction (Table 1). The reactions were performed and measured using a BioRad MyIQ detection system (BioRad) with SYBR green fluorophore. Relative target gene expression was normalized to a set of eight reference genes (tested in Genorm with a pairwise variation (PV) of 0.07) for the transfection experiments. For the cluster analysis, 6 reference genes were used with a PV 0.07 (HNRPH, TBP, SRPR, HMBS, RPS5 and RPS19). A relative induction of gene expression was statistically assessed using paired, 2-tailed student’s *T*-test. Relative expression was calculated by the delta-delta Ct (ΔΔCt) method [23].

**Table 1:** Primers used

OMIM	Symbol	Forward primer	Reverse primer	Annealing (°C)	Size (bp)	Accession number
Target genes						
CTNNB1	β-Catenin	ATGGGTAGGGCAAATCAGTAAGAGGT	AAGCATCGTATCACAGCAGGTTAC	64.0	106	XM_005634157.1
AXIN2	Axin2	GGACAAATGCGTGGATACCT	TGCTTGGAGACAATGCTGTT	60.0	141	XM_548025
BNIPL	BCL2	TGGAGAGCGTCAACCGGGAGATGT	AGGTGTGCAGATGCCGGTTCAGGT	62.0	87	AY_509563.1
CCND1	CYCLIND1	GCCTCGAAGATGAAGGAGAC	CAGTTTGTTCACCAGGAGCA	60.0	117	NM_001005757.1
ERBB1	EGFR	CTGGAGCATTCGGCA	TGGCTTTGGGAGACG	53.0	107	XM_533073
ERBB2	HER2	CGTGCTGGACAATGGAGACC	CCGCTGAATCAAGACCCCTC	64.0	51	AB008451
ERBB3	HER3	TAGTGGTGAAGGACAACGGCAG	GGTCTTGGTCAATGTCTGGCAG	70.0	103	XM_538226
HSP90AA1	HSP90	CTTGACCGATCCCAGTAAGC	TATTGATCAGGTCGGCCTTC	59.0	127	XR_134513.2
ID1	ID1	CTCAACGGCGAGATCAG	GAGCACGGGTTCTTCTC	59.5	135	XM_847117.2
ID2	ID2	GCTGAATAAATGGTGTTCGTG	GTTGTTCTCCTTGTGAAATGG	60.5	114	XR_134413.1
TCF7L3	LEF1	AGACATCCTCCAGCTCCTGA	GATGGATAGGGTTGCCTGAA	60.0	137	XP_863334.2
MET	cMET	TGTGCTGTGAAATCCCTGAATAGAATC	CCAAGAGTGAGAGTACGTTTGGATGAC	56.0	159	NM_001002963.1
MUC1	MUC1	CTATGAGGAGGTTTCTGCAG	GAACACAGTTGAGAGGAGAG	62.0	172	NM_001194977
NCOA3	NCOA3	ATGCGGCCTGGTGAGATT	TAAGAAGTGGCCTATTTTGAGTCC	67.1	141	ENSCAFT00000017243
PTEN	PTEN	AGATGTTAGTGACAATGAACCT	GTGATTTGTGTGTGCTGATC	62.0	102	NM_001003192.1
RAC1	RAC1	TCCCTTATCCTATCCGCAAA	ATGATAGGGGTGTTGGGACA	58.0	128	NM_001003274.2
RAC1B	RAC1b	TGGGATACAGCTGGACAAGA	CTTGTCTTTGCCCCTGGAG	58.0	108	JN_182651.1
SNAI2	SLUG	CTTCACTCCGACTCCAAACG	TGGATTTTGTGCTCTTGCAG	60.0	147	XM_005637933.1
Reference genes						
HMBS	HMBS	TCACCATCGGAGCCATCT	GTTCCCACCACGCTCTTCT	61.0	112	XM_546491
HNRNPH2	HNRPH	CTCACTATGATCCACCACG	TAGCCTCCATAACCTCCAC	61.2	151	XM_538576
HPRT1	HPRT	AGCTTGCTGGTGAAAAGGAC	TTATAGTCAAGGGCATATCC	57.0	104	NM_001003357
RPS5	RPS5	TCACTGGTGAGAACCCCCT	CCTGATTCACACGGCGTAG	62.5	141	XM_533568
RPS19	RPS19	CCTTCCTCAAAAAGTCTGGG	GTTCTCATCGTAGGGAGCAAG	62.0	95	XM_533657
SRPR	SRPR	GCTTCAGGATCTGGACTGC	GTTCCCTTGGTAGCACTGG	61.2	81	XM_546411
TBP	TBP	CTATTTCTTGGTGTGCATGAGG	CCTCGGCATTCAGTCTTTTC	57.0	96	XM_849432
YWHAZ	YWHAZ	CGAAGTTGCTGCTGGTGA	TTGCATTTCCTTTTTGCTGA	58.0	96	XM_843951

### Cell viability

Cell viability was determined by means of the colorimetric 3-[4,5-dimethylthiazol-2-yl] 2,5-diphenyltetrazolium bromide assay (MTT) (Sigma Aldrich, Zwijndrecht, The Netherlands). Briefly, cells were seeded in 96 wells plates (Primaria, BD Biosciences, Breda, The Netherlands) and after 24 h incubation to attach and stretch, treated with the different compounds for 40 h. Cell viability was determined by incubating 20 μl 5 mg/ml MTT in 100 μl medium in each well. After a 2 h incubation, the media was removed by decanting and 100 μl DMSO was added to each well, incubated for 30 min and the absorbance was measured at 595 nm in a spectrophotometer Anthos Multimode Detector (Anthos Mikrosystem GmbH, Krefeld, Germany). IC50 curves were plotted with Sigma-plot version 12.5.

### Protein extraction and Western blot

Cells were seeded in 75 cm^2^ bottles and after 24 h incubation to attach and stretch, treated with the different compounds. After 40 h cells were washed with cold HANK’s balanced salt solution and lysed and scraped with RIPA buffer [5]. After 20 min incubation on ice, samples were centrifuged for 15 min at 16,000 g and 4 °C. Protein concentration was determined using Bio-Rad Dc Protein Assay (Bio-Rad Laboratories). Twenty micrograms of protein from total cell lysates was subjected to SDS-PAGE and analyzed by Western blot. Primary antibodies used in this study were directed against β-Catenin (Ab6302 1:4000) (Abcam, Cambridge, UK), HER2 (PA5-14635 1:500) (Pierce-Thermo Scientific), HER3 (PA1-86644 1:2500 (Thermo Scientific), with β-Actin pan Ab-5 (MS-1295-P1 1:2000) (Thermo Scientific) as a reference protein. And as secondary antibody, goat anti-mouse HRP-conjugated (HAF007), goat anti-rabbit HRP conjugated (HAF008) and donkey anti-goat HRP conjugated (HAF109) (R&D Systems, Abingdon, UK) was used in a 1:20.000 dilution. HRP was visualized using Advance TM_Enhanced chemiluminescence (ECL, Amersham, GE Healthcare, Eindhoven, The Netherlands) and analyzed using GelDoc 2000 (Bio Rad). With the Quantity One software, version 4.6.9 (BioRad), densities were measured, corrected for the background and related to β-Actin expression as loading control.

### Statistics

Cluster analysis with qPCR results from all the cell lines was done in RStudio (version 3 software (R Core Team (2013) R: A language and environment for statistical computing. R Foundation for Statistical Computing, Vienna, Austria; http://www.R-project.org)) with a Pearson:Spearman test (genes: Pearson; samples: Spearman average) to find a correlation between the TOP/FOP ratio, related target genes and the cell lines.

Transfection and incubation studies, and also the MTT assays, were done in three independent experiments (*n* = 8 for MTT and *n* = 4 for each transfected/incubation compound). After background subtraction, the cell viability, the Wnt-signaling and the protein levels were calculated as a percentage of the non-treated cells. IC50 curves were done in a single experiment with *n* = 8 and the HER3 Western blots were done in triplicate. Differences in TOP/FOP activities, RNA, cell viability and protein expression levels were statistically assessed using unpaired, two tailed Student’s *t* test, a P value less than 0.01 was considered significant.

## Results and discussion

### Wnt activity and gene clustering

**Fig. 1:**
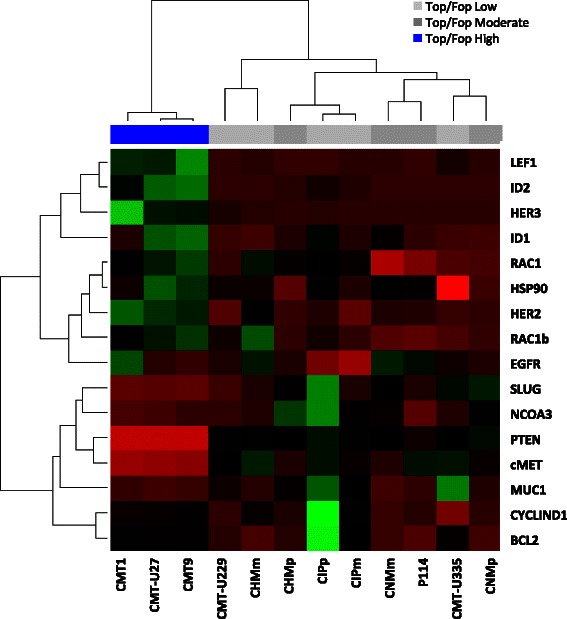
Cluster analysis of different canine mammary cell lines with their relative basal gene expression. RNA from 12 different canine mammary cancer cell lines was isolated and quantitative RT-PCR was done for several target and reference genes on their cDNA. RT-PCR data were analyzed in RStudio with the Pearson:Spearman test and graphically presented with their corresponding TOP/FOP ratios (blue >30; dark grey 2-6; light grey <2) as published previously [5]. The three cell lines with high basal TOP/FOP ratios (canonical Wnt pathway) cluster together in the non-hierarchical cluster analysis. Green is a high expressed gene and red is low expressed gene

In a continuation of our previous findings of a variation in basal Wnt/β-Catenin activity in canine mammary tumor cell lines [5] we searched for the mRNA expression of members of the EGF receptor family of proteins and the associated signal-transduction pathways. In the present paper, the three cell lines with high ligand-independent basal Wnt activity (CMT1, CMT-U27 and CMT9) were, through cluster analysis of the gene expression data, shown as a clear grouping, but no clear association for absent or low Wnt-ligand induced basal Wnt/β-Catenin activity in the other cell lines (Fig. 1). High basal Wnt activity is clearly grouped together with an up-regulated expression of *LEF1*, *ID1*, *ID2*, *EGFR* (only in CMT1*)*, *HER2/3*, *HSP90* and *RAC1* mRNA and a down regulation of *SLUG, NCOA3, MUC1, CYCLIN D1, BCL2, cMET* and *PTEN* mRNA. In these highly activated Wnt cell lines, *PTEN* expression is completely lost. PTEN deficiency occurs in 5 to 14 % of primary human breast cancers and *PTEN* is also lost in human T47D and MCF7 cell lines [13]. The cluster analysis confirmed our previous finding that in the cell lines with active Wnt signaling, *LEF1* is overexpressed and acts as a contributing factor of the high canonical Wnt pathway [5]. New is our finding of a strong association of the expression of the EGF pathway both at the receptor level (*HER* expression) as in the silencing of *PTEN* with high ligand independent Wnt signaling. Previously, it has been shown that binding of Wnt proteins to the Frizzled receptor may activate the EGFR. EGFR can be further activated by homo or hetero-dimerization with HER2, HSP90 as its chaperone, or HER3. These activated complexes functions as an oncogenic unit to drive the proliferation of breast cancer cells [14, 24]. In human and mouse mammary glands, the activated Wnt/β-Catenin is related to enhanced *HER2* expression [25], something also found in 30 % of canine malignant mammary tumors [26]. Combining the up regulation of both *HER2* and *HER3* in our canine mammary cell lines and the fact that the HER2/HER3 complex is the strongest activator for downstream signaling pathways makes this an interesting area for further research [15, 27, 28].

We concluded that the association between high Wnt signaling (high TOP/FOP ratios), high *LEF1* levels, loss of *PTEN* and high *HER2/3* mRNA levels all point to the activation of the PI3K/AKT/mTor pathway [29–32]. We therefore investigated the effects of PI3K/mTor inhibitors on cell viability, Wnt signaling, expression levels of the differently expressed genes from the clustering analysis and the cellular content of β-Catenin, and HER2/3 protein as an experimental model for HER2+/PTEN- breast cancer.

### PI3K/mTor inhibition decreases cell viability but stimulates Wnt activity

**Fig. 2:**
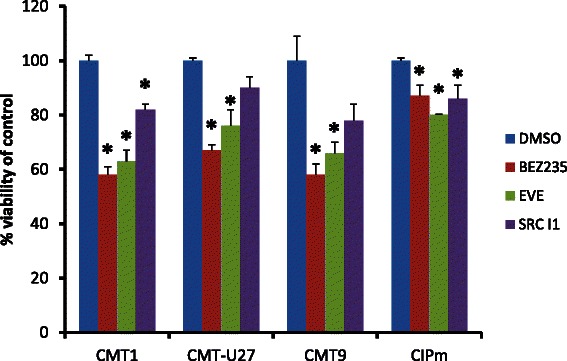
Effects of drugs on cell viability. Cell viability (MTT assay) was measured after treatment with PI3K/mTor inhibitor BEZ235, mTor inhibitor Everolimus and cSRC inhibitor Src-l1. Three cell lines with a high TOP/FOP ratio (>30) (CMT1, CMT-U27, and CMT9) and CIPm cells with a low TOP/FOP ratio (<2) were grown for 40 h in the presence of PI3K/mTor inhibitor BEZ235 (50nM), mTor inhibitor Everolimus (100nM) and cSRC inhibitor Src-l1 (20 μM). After 40 h the cell numbers were measured with a MTT assay. Results expressed as % of control are the mean (±STDEV) of three independent experiments. *P < 0.01 versus appropriate cell line control (DMSO)

Cell lines with high Wnt activity and a cell line lacking basal Wnt activity (CIPm) were incubated with Everolimus as a specific mTor inhibitor and BEZ235 as a dual inhibitor of PI3K and mTOR. Both BEZ235 and Everolimus equally inhibited the viability of the highly activated Wnt cell lines and have only a slightly inhibitory effect on the CIPm cells (Fig. 2).

**Fig. 3:**
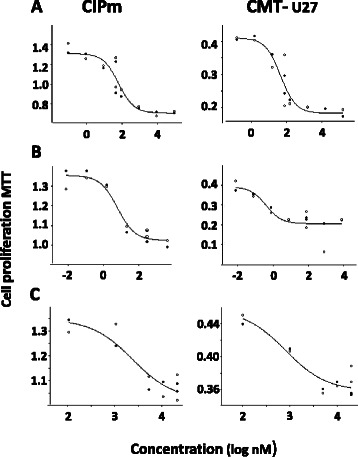
IC50 curves. CMT-U27 (high TOP/FOP ratio, >30) and CIPm cells (low TOP/FOP ratio, <2) were grown for 40 h with the three inhibitors, BEZ235 (**a**), Everolimus (**b**) and Src-l1 (**c**) in increasing concentrations (BEZ235 0.1nM–100 uM, Everolimus 0.01 nM–10 μM and Src-I1 0.1 μM–20 μM). After 40 h the cell viability was measured with a MTT assay. Results, expressed as % of control, are the mean (±SEM) of three independent experiments. *P < 0.01 versus appropriate cell line control (DMSO). IC50 values were calculated with Sigma-Plot software (version 12.5)

**Fig. 4:**
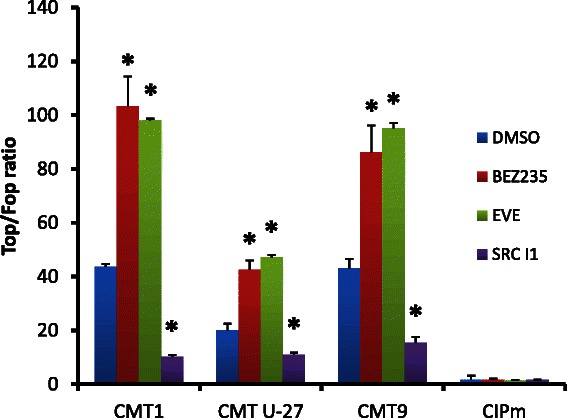
Effects of drugs on Wnt signaling. PI3K/mTor inhibitor BEZ235 and mTor inhibitor Everolimus stimulates the TOP/FOP ratios. Incubation with the cSRC inhibitor Src-l1 shows a down regulation of the Wnt reporter activity. Three cell lines with a high TOP/FOP ratio (>30) (CMT1, CMT-U27, and CMT9) and CIPm cells with a low TOP/FOP ratio (<2) were grown for 40 h in the presence of PI3K/mTor inhibitor BEZ235 (50nM), mTor inhibitor Everolimus (100nM) and cSRC inhibitor Src-l1 (20 μM). After 40 h the TOP/FOP ratio was measured with a Dual-Luciferase assay. The mean ratio is expressed of three independent experiments (±SEM). *P < 0.01 versus appropriate cell line control (DMSO). PI3K/mTor inhibitor BEZ235 and mTor inhibitor Everolimus stimulates the TOP/FOP ratios. Incubation with the cSRC inhibitor Src-l1 shows a down regulation of the Wnt activity in the basal activated Wnt cell lines

This inhibitory effect on viability was clearly dose-dependent and present in both the high basal Wnt cell line CMT-U27 and the CIPm cell line (Fig. 3 and Table 2). Comparable concentrations of BEZ235 were also used in human studies with different cell lines [33, 34]. In human MCF7 cells, Everolimus (Rad001) was tested in a range from 0-1000 nM and only the 1000 nM concentration showed a 50 % reduction in cell number [35]. Incubation of these cells with BEZ235 and Everolimus resulted in unexpected further significant enhancement of Wnt activity of CMT1, CMT-U27 and CMT9, with a more than 2-fold increase in the TOP/FOP ratios (Fig. 4).

**Table 2:** IC50 values for inhibition of cell proliferation

Inhibitor	IC50 proliferation		Concentration used	References
	CIPm	CMT -U27		
BEZ235	69 nM	29 nM	50 nM	[33, 34]
Everolimus	3 nM	1 nM	100 nM	[35]
Src-I1	4 μM	1 μM	20 μM	[49]

BEZ235-induced inhibitions of cell proliferation have been reported previously in MCF-7/shPTEN cells and in 21 other tested human cancer cell lines [13]. The only difference is that in these cell lines, BEZ235 had a stronger effect than Everolimus on cell proliferation [36]. In human breast cancer, loss of PTEN is associated with activation of the PI3K/AKT Pathway [12], which may result in increased nuclear stabilization of β-Catenin in mammary stem cells using human breast tissue xenografts [37]. This is in contrast to the absence of any inhibitory effect in the TOPFlash reporter assays of several breast and prostate cancer cell lines treated with another inhibitor of the PI3K pathway (Wortmannin) and suggests that the PI3K pathway has no relation with Wnt-mediated transcriptional activity [30]. In our cell lines as well, neither Wortmannin nor the introduction of a PTEN-expressing plasmid inhibited Wnt signaling, indicating that PI3K inhibition alone does not influence the active Wnt signaling and that mTor inhibition is needed (data not shown). CIPm, which lacks basal Wnt activity, did not respond to both inhibitors with increased reporter activity.

We conclude that inhibition of PI3K and/or mTor in the highly activated Wnt cell lines inhibits cell viability, but further stimulates Wnt activity. Because the mechanism behind this remained unclear, we tested the effect of these inhibitors on the expression of genes from the cluster analysis.

### PI3K/mTor inhibitors stimulate *β-Catenin*, *Axin2*, *MUC1*, *cMET*, *EGFR* and *HER2* expression in the activated Wnt cell lines

**Fig. 5:**
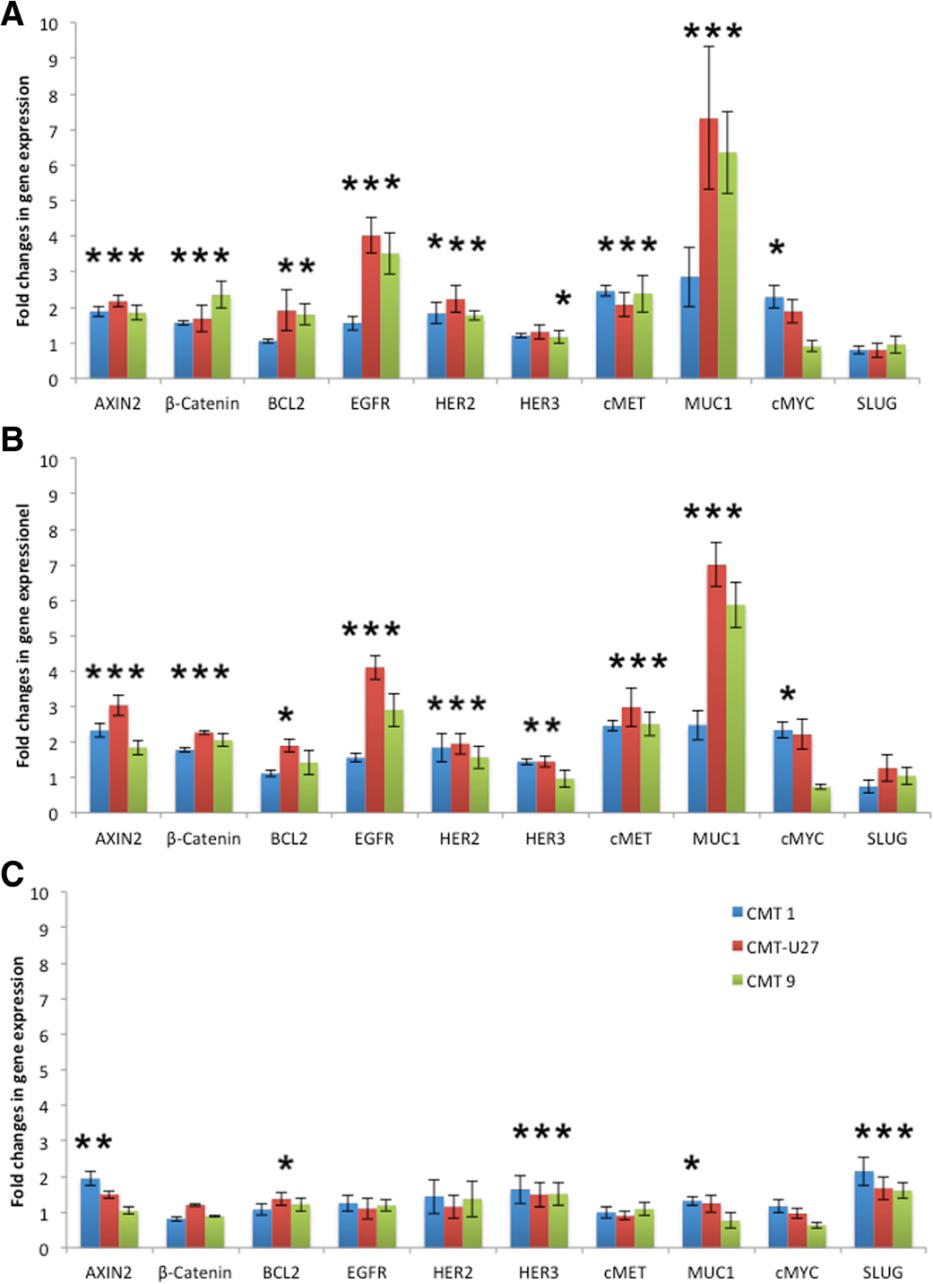
Effects on gene expression. CMT1, CMT-U27 and CMT9 cells were grown for 40 h with the three inhibitors, 50nM BEZ235 (**a**), 100nM Everolimus (**b**) and 20 μM Src-l1 (**c**). After inhibition RNA was isolated and the relative expression of several target genes was measured by quantitative RT-PCR. The differences in expression levels compared to an appropriate control were expressed as the mean of 12 samples out three independent experiments. *P < 0.01

**Fig. 6:**
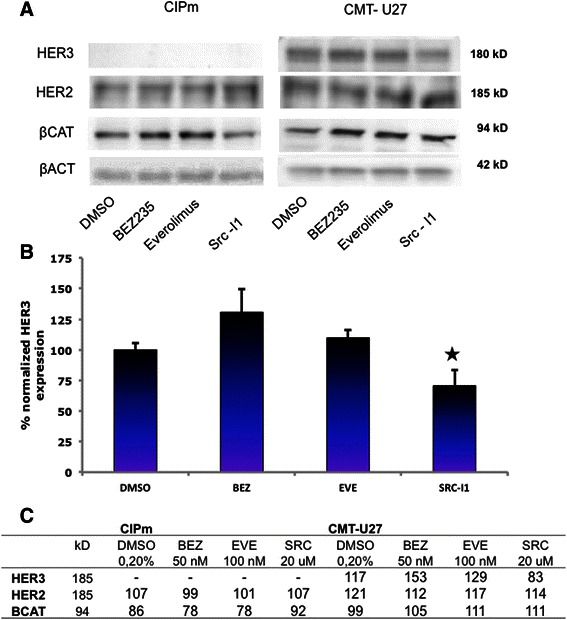
Protein levels in CIPm and CMT-U27 inhibited with BEZ235, Everolimus and Src-I1. Cells were cultured for 40 h with the inhibitors, 50nM BEZ235, 100nM Everolimus and 20 μM Src-l1. Total protein was isolated with RIPA buffer and 20 μg protein was used for Western Blot analyses. Blots were probed with total antibodies for β-Catenin, HER2, HER3 and β-Actin (**a**). The HER3 blot was done in triplicate and normalized against β-Actin. Results, expressed as % of control are the mean (±STDEV) *P < 0,05 versus DMSO control (**b**). Densities were measured, corrected for the background and related to β-Actin expression as loading control, expressed in % (**c**)

Inhibition of the PI3K/mTor pathway with BEZ235 (a dual PI3K mTor inhibitor) or with Everolimus (a single mTor inhibitor) in cell lines with high ligand-independent canonical Wnt activity resulted in comparable upregulation of a number of candidate genes (Fig. 5). A moderate twofold increase was detected in the expression of *β-Catenin, Axin2*, *HER2* and *cMet* mRNA*.* Only the expression of *EGFR* and *MUC1* mRNA was higher, especially in the CMT-U27 and CMT9 cell lines. β-Catenin and Axin2 are markers for an activated Wnt/β-Catenin signaling in mammary tissues [38]. Protein levels of β-Catenin were not significantly different after treatment with both inhibitors (Fig. 6). The stable total protein expression of β-Catenin may reflect predominantly binding to E-cadherin, which is comparable high in both cell lines [5, 7] and may not be related to the cytoplasmic or nuclear ß-Catenin content. No significant effect of both inhibitors was seen on HER2/3 protein content although HER3 expression was only found in the cell line with high basal Wnt activity (Fig. 6). Apart from the expression of HER2 at the cell surface it can also form a complex with AP-1/Stat3/PR in the nucleus. This nuclear HER2 can act as a transcription factor and modulate genes involved in breast cancer proliferation [9]. In contrast with the absence of HER3 in the CIPm cell line, the CMT-U27 cells have a HER3 band visible at 180kD, which correspond to a glycosylated form. This glycosylated HER3 protein is also found in human MDA-MB-445 and MDA-MB-453 cells [39]. In MDA-MB-453 and other mammary tumor cell lines, there is strong evidence for the role of HER3 in cancer. HER3 plays a role in EMT and activated HER3 can also increase the expression of the cMET proto-oncogene independently from EGFR and HER2 [16], which may be in line with the 2-3 fold increased expression shown in Fig. 5. *EGFR* and *HER2* expressions are upregulated after treatment with BEZ235 in a human breast cancer cell line with a lack of PTEN (MDA-MB-468). In contrast with this *BCL2* is not upregulated in all tested canine cell lines and *HER3* is only, although significantly, 1.5 times upregulated in some cases [40]. The most prominent change was the 7-fold increase in *MUC1* expression. At the same time, in all tested cell lines, basal MUC1 protein concentrations showed no difference, even in the presence of considerable amounts of the cytoplasmic domain MUC1-CD (data not shown). Still, overexpression of MUC1 plays a role in tumor formation and progression in > 90 % of the breast cancers [41]. It has been reported that the Wnt activity is directly affected through the binding of the Wnt effector β-Catenin on MUC1-CD [42] through binding of several proteins, including cMET, cSRC and EGFR/HER. Consistent with this is the enhanced *EGFR* and oncoprotein *MUC1* mRNA concentrations in our experiment together with the upregulation of cMET. Enhanced *cMET* expression has been linked to poor prognosis in human breast cancer, since it influences extravasation/angiogenesis, enhances resistance to endocrine therapy and trastuzumab treatment and may influence the regulation of β-Catenin/TCF transcription [29, 43]. The basal expression of *cMET* in the cell lines with elevated Wnt activity is low, but stimulated when the PI3K/mTOR pathway is blocked; blocking this pathway also affects the *EGFR* and *HER2* expression. Overexpression of MUC1 is associated with the induction of anchorage-independent growth and tumorigenicity in human carcinomas. *MUC1* overexpression is also directly associated with stabilization of β-Catenin either via MUC1 binding to E-Cadherin, or by inhibition of the adenomatous polyposis coli (APC)/GSK3β/ MUC1-CD complex, whereas phosphorylation of MUC1-CD by EGFR and cSRC increases β-Catenin [42, 44–46].

Leaving aside the question of how EGFR/HER2/HER3 signaling is involved in high basal Wnt activity, it is clear that the upregulation of the MUC1 and EGFR/HER2 expression have a relationship with the increased Wnt activity that occurs after inhibition of the PI3K/mTor pathway. The presence of glycosylated HER3 protein in the highly activated Wnt cell line is new, and because our results suggest that activation of cSRC could play a role [15, 46], we next studied the influence of cSRC inhibitors on the Wnt activity and HER3 levels.

### Active Wnt signaling significantly inhibited by the cSRC inhibitor Src-I1

Activation of EGFR initiates the activation of PI3K/AKT/mTor and MAPK as well as various other signaling pathways, including the PLC-Υ1, JAK/STAT, and cSRC pathway [15, 47]. Because the EGFR can form a complex with cSRC, FAK (focal adhesion kinase), integrins and β-Catenin resulting in growth, viability, survival and migration [46], we tested the effect of the cSRC inhibitor 1 (Src-I1) and a FAK inhibitor [48, 49]. Treatment with the FAK inhibitor resulted in a small non-significant decreases in the TOP/FOP ratio with no effect on cell proliferation (data not shown), however Src-I1 had a clear effect on cell viability and was found to be an inhibitor of the Wnt activity via inhibition of cSRC signaling [50, 51]. The same inhibition on the Wnt pathway was found in the human cell lines NIH 3 T3 with the cSRC inhibitor PP1 [52]. The used concentration of Src-I1 reduced cell proliferation for some 20 % and acted as a weaker inhibitor in comparison with BEZ235 and Everolimus (Fig. 2). Unlike BEZ235 and Everolimus, the cSRC inhibitor Src-I1 reduced the Wnt/β-Catenin signaling by almost half in canine mammary tumor cell lines with high canonical Wnt activity, but not in the CIPm cells that lack basal Wnt activity (Fig. 4). Inhibiting mouse cells with cSRC inhibitors PP2 and Genistein gave comparable results on LEF/TCF sensitive transcription and on the level of activated phosphorylated cSRC. This indicates that cSRC is a positive regulator of the Wnt/β-Catenin signaling [50]. To investigate the Wnt-regulation by both the PI3K pathway and the cSRC pathway, we tested the same target genes used in the clustering analyses (Fig. 1).

### Effects of cSRC inhibition on gene and protein expression

The cSRC inhibitor Src-l1 decreased the cell viability (Fig. 2), decreased Wnt signaling (Fig. 4) without affecting *MUC1, EGFR* and *HER2/*HER2 expression levels (Figs. 5 and 6). The inhibition of Wnt activity as measured by a decrease in TOP/FOP ratios was also clearly dose-dependent (data not shown) in the activated Wnt cells. Marginal increases were found in *SLUG* and *HER3* mRNA concentrations (Fig. 5c) after cSRC inhibition. Remarkably inhibition with Src-I1 showed a significant decrease in HER3 protein concentrations whereas the HER3 mRNA expression was slightly elevated indicating that cSRC inhibition is associated with a higher turnover or a decreased glycosylation of HER3 at the protein level. At the moment information on the regulation of *HER3* expression is scarce. Several microRNAs regulate the expression levels and also epigenetic effects have been shown to influence *HER3* expression in breast cancer cells [16].

## Conclusions

We see an association between the Wnt pathway and the EGFR/HER2/HER3 stimulated cSRC (motility) route. The role of MUC1 is less clear and needs further research. In mice, however, data are available showing that MUC1 activates cSRC signaling by influencing the association of PI3K and β-Catenin leading to a delay in mammary tumor progression [46]. Prior research indicates that activation of the PI3K/mTor pathway may be related to resistance to hormonal therapies. Administration of the mTor inhibitor Everolimus has therefore been studied extensively in women with HR+, HER2+ or TNBC breast cancer. Reports on various studies, i.e. BOLERO (Breast Cancer Trials of Oral Everolimus), TAMRAD and EFECT, have shown an increased progression free survival (PFS) when Everolimus was combined with aromatase inhibitors [53]. If the effects shown in this paper on the *in vitro* inhibition of cell viability but increased Wnt signaling are also present in a subset of breast cancer patients then treatment with Everolimus could have an adverse effect on Wnt regulated aspects such as stemness and metastasis.

cSRC may play a crucial role in tumor growth and metastasis. Metastasis involves loss of cellular adhesion, increased motility, intravasation, invasion, extravasation, resistance to anoikis, and colonization at distant sites. High cSRC expression is found in various breast cancers in association with reduced survival. Recently the cSRC inhibitors Dasatinib and Bosutinib were studied as a monotherapy in humans, but showed no clear reduction of antitumor activity, indicating that before further studies are done, clear biomarkers should be found for proper patient selection [54]. The search for these biomarkers is being aided by research on dogs with spontaneous mammary carcinomas. For example, the successful treatment with Dasatinib of a dog with osteosarcoma, reported in a recent case study, shows that the dog is an interesting animal for further clinical studies using cSRC inhibitors in HER2+/PTEN- tumors [26, 55, 56]. Binding of cSRC to HER2 is crucial for the formation of HER2:HER3 heterodimers. Both phosphorylated and total levels of cSRC are upregulated in anti-estrogen resistant T47D cell lines that show growth reduction by cSRC inhibitors. EGFR homodimers also play an important role in growth. The individual contributions of EGFR, HER2 and HER3 in Wnt pathway activation require further study. Our results on highly activated Wnt signaling cells require further research into the role of HER3, cSRC, and Wnt on tumor growth and metastasis [57].

In summary, inhibition of the PI3K/mTor pathway in tumor cell lines with ligand-independent Wnt activity further increases canonical Wnt signaling. As we have shown, this Wnt activity can be effectively inhibited by a cSRC inhibitor. While very recently, loss of PTEN has been associated with worse outcomes in HER2 positive breast cancer patients, no data are available on their Wnt activity [58]. Although a number of human breast cancer cell lines have active ß-catenin signaling (summarized in [5]), this relation between HER2/3 expression and PTEN loss appears not to have been previously documented.

Further studies will need to identify optimal biomarkers for selecting breast cancer patients most likely to benefit from concerted treatment with combination therapies of mTor and cSRC inhibitors. Such research is very likely to include studies in dogs with spontaneous mammary carcinomas.

## Abbreviations

AP1: Activator protein 1
APC: Adenomatous polyposis coli
cMET: Met proto-oncogene
cSRC: Rous sarcoma proto-oncogene
E2: Estradiol
EGF: Epidermal growth factor
EGFR: EGF receptor
FAK: Focal adhesion kinase
GSK3ß: Glycogen synthase kinase 3 beta
HER2/3: Human epidermal growth factor receptor 2/3; ERBB2/3
HSP90: Heat shock protein 90
ID1/2: Inhibitor of DNA binding 1/2
JNK: Jun N-terminal kinase
LEF1: Lymphoid enhancer-binding factor 1
MAPK: Mitogen-activated protein kinase
mTor: Mammalian target of rapamycin
MUC1: Mucin 1
NFAT: Nuclear factor of activated T-cells
P4: Progesterone
PI3K: Phosphatidyl-3-kinase
PTEN: Phosphatase and tensin homolog
RAC1: Ras-like protein
TCF: T-cell-specific transcription factor
Wnt: Wingless-type MMTV integration site family
